# Exploring the genetics of feed efficiency and feeding behaviour traits in a pig line highly selected for performance characteristics

**DOI:** 10.1007/s00438-017-1325-1

**Published:** 2017-05-12

**Authors:** Henry Reyer, Mahmoud Shirali, Siriluck Ponsuksili, Eduard Murani, Patrick F. Varley, Just Jensen, Klaus Wimmers

**Affiliations:** 1 Leibniz Institute for Farm Animal Biology, Institute for Genome Biology, Wilhelm-Stahl-Allee 2, 18196 Dummerstorf, Germany; 2 Department of Molecular Biology and Genetics, Center for Quantitative Genetics and Genomics, Aarhus University, 8830 Tjele, Denmark; 3 Hermitage Genetics, Sion Road, Kilkenny, Ireland

**Keywords:** Feed efficiency, Feeding behaviour, Pigs, GWAS, FCR

## Abstract

The consideration of feed efficiency traits is highly relevant in animal breeding due to economic and ecologic impacts of the efficient usage and utilization of feed resources. In pigs, corresponding observations are recorded using automatic feeding stations and serve as one of the main criteria in most pig selection programmes. Simultaneously, feeding stations also generate feeding behaviour data which represent a nearly unused resource and provide a valuable proxy measure of health status, animal welfare, and management practices. In the current study, an integrated approach was applied to a feed efficiency tested and genome-wide genotyped terminal sire line population. Therefore, genetic analyses were performed combining a single-marker based approach and a Bayesian multi-marker algorithm. Major quantitative trait loci (QTL) for feeding behaviour traits comprising daily occupation time, daily feeder visit, and daily feeding rate were identified on chromosomes 1, 4, 6, 7, 8, and 14. Feed efficiency was represented by feed conversion ratio and daily feed intake revealing prominent genomic regions on chromosomes 1, 6, 9, and 11. The positional and functional candidate genes identified are involved in transport processes like *AQP4*, *SLC22A23*, and *SLC6A14* as well as energy sensing, generation, and utilization as exemplified by *PPP3CA*, *IQGAP3*, *ECI2*, and *DnaJC15*. These molecular features provide the first step towards the dissection of the genetic connection between distinct feeding behaviour patterns, feed efficiency and performance, health, and welfare traits driving the implementation of these traits in breeding programmes and pig husbandry.

**Electronic supplementary material:**

The online version of this article (doi:10.1007/s00438-017-1325-1) contains supplementary material, which is available to authorized users.

## Introduction

Beside growth rate and lean meat percentage, feed efficiency (FE) is the most important selection criteria implemented in breeding programmes affecting economic aspects and the environmental footprint of pig meat production (Kanis et al. 2005; Reckmann et al. 2016). Feed conversion ratio (FCR) is expressed as the ratio of body weight gain and feed intake. In practice, it is usually recorded for selected pigs during the grower–finisher phase via automated feeding stations implemented in group-housing systems (Maselyne et al. 2015). An increased importance has been attached to FE traits in animal breeding to consider the efficient conversion of nutrients into body mass and as a major factor driving the productivity and the profitableness of the production system (Arthur and Herd 2005). Thus, FE and related traits are a major target for genomic selection strategies in livestock breeding (current state of research summarized by Samorè and Fontanesi 2016). The variation in FE is related to extrinsic factors like the formulation and energy density of diets, management, and climatic conditions. Intrinsic factors are mediated by diverse physiological processes including sensory and cerebral regulation of appetite, gut digestibility, nutrient absorption, thermoregulation, muscle activity, as well as processes related to anabolic and catabolic metabolism (reviewed by Herd and Arthur 2009). Major factors with contributions to underlying signalling cascades are involved in the gut–brain axis as depicted for instance by the neuropeptides ghrelin, peptide YY, cholecystokinin, and leptin (van der Klaauw and Farooqi 2015). Despite these overall biological factors that control appetite and satiety, little is known about the molecular connections between FE and feeding behaviour traits and its genetic and phenotypic correlations. In fact, both groups of traits are influenced by environmental and genetic factors (Kallabis and Kaufmann 2012; Maselyne et al. 2015; Shirali et al. 2015). Specifically, Fernández et al. (2011) provided evidence for the occurrence of pig breed-specific feeding strategies substantiating the influence of genetics on these traits. Accordingly, Do et al. (2013) revealed breed-specific differences in heritability estimates of FE and feeding behaviour traits as well as variations in their phenotypic and genetic correlations among three different Danish pig breeds. Feeding behaviour traits like daily feeding rate, daily time spent eating, or daily feeder visits were previously characterised as moderately to highly heritable (Chen et al. 2010; Do et al. 2013). The individual differences in feeding behaviour traits based on genetic factors provide a source of valuable molecular biomarkers to forecast feeding behaviours as well as their implementation in pig husbandry (Brown-Brandl et al. 2013). Specifically, feeding behaviour observations could be used as an automated tool to monitor the health status of animals towards improved disease detection and to assess the management practice (Weary et al. 2009; Brown-Brandl et al. 2013).

The objective of this study was to elucidate genetic factors affecting FE and feeding behaviour traits in a terminal boar population in which each animal was genotyped on a genome-wide scale for ~60 K single-nucleotide polymorphisms (SNPs). Investigated traits comprise FCR, daily feeder occupation time (DOT), daily feed intake (DFI), daily feeder visit (DFV), and daily feeding rate (DFR). Single- and multi-marker genome-wide association studies (GWAS) were performed to identify both trait-specific and overlapping quantitative trait loci (QTL).

## Materials and methods

### Animal care statement

The experiment was conducted under experimental licence from the Irish Department of Health. Animal handling and treatment was in accordance with the Cruelty to Animals Act 1876 and the 1994 European Communities Regulations (Amendments of the Cruelty to Animals Act 1876).

### Housing and feeding

Boars of the terminal Maxgro line were reared, housed, and sampled by Hermitage Genetics (Kilkenny, Ireland). The Maxgro line is predominately Pietrain based and under continuous selection for feed conversion efficiency, growth performance, and leanness. For testing of FE and feeding behaviour traits, pigs were penned on the basis of initial body weight (mean ± SD 53.4 ± 10.6 kg) and assigned to the same dietary treatment. Ingredients and composition of the diet are provided in Table 1. Average ages of pigs at start and end of the test period were 97.9 ± 9.8 and 146.0 ± 9.3 days, respectively. The end of the test period was determined by reaching a final body weight of 110 kg (mean ± SD 114.6 ± 9.8 kg). Groups of 14 animals were housed in fully slatted pens with a space allowance of 0.75 m^2^ per pig. The house was mechanically ventilated to provide an ambient temperature of 18 °C. Pens were equipped with single-space computerised feeders (Mastleistungsprüfung MLP-RAP; Schauer Agrotronic AG, Sursee, Switzerland) as described by Varley et al. (2011). Via ear-tag transponders, individuals were registered to automatically record the individual amount of consumed feed per feeder visit as well as the entry and exit times. Pigs had ad libitum access to feed and water.

**Table 1:** Ingredient and nutritional composition of finisher diets

Ingredient composition (%)		Nutrient composition	
Barley	50.00	Protein (%)	16.54
Maize	10.00	Oil (%)	3.21
Wheat	18.20	Fibre (%)	3.57
Hipro soya	17.40	Ash (%)	4.80
Soya oil	1.40	DE (MJ/kg)	13.78
Mono dicalcium phosphate	0.90	NE (MJ/kg)	9.90
Finisher premix^a^	2.10		

^a^Premix provided per kg of complete diet: 10,000 IU vitamin A, 2000 IU vitamin D_3_, 100 IU vitamin E, 10 mg anti-oxidant mix, 150 μg biotin, 15 mg copper, 100 mg zinc, 2 mg iodine, 0.35 mg selenium, and 100 mg iron

### DNA extraction and genotyping

At the end of the test period, blood samples were taken from the *Vena jugularis* using EDTA as anticoagulant. DNA was extracted employing the QIAamp DNA Blood Mini Kit (Qiagen, Hilden, Germany) following the manufacture’s recommendations. In total, samples of 846 boars with observations of FCR and feeding behaviours were processed and subsequently genotyped using porcine SNP60 Beadchips on an iScan system (Ramos et al. (2009); Illumina, San Diego, CA, USA). All sample showed sample call rates above 0.97. For each sample, information of 60487 markers was retrieved after removing all SNPs with call frequencies below 0.95. Of these markers, 59070 SNPs mapped to the *Sus scrofa* genome build 10.2 (http://www.animalgenome.org/repository/pig/, issued 2014-07-07) which includes all 18 autosomes, both sex chromosomes, and a contig of unmapped markers (UWGS). Gaps in the genotype matrix were closed via imputation of missing SNP information using fastPHASE (v1.2) (Scheet and Stephens 2006). After filtering for minor allele frequency (MAF ≥ 0.03), 51,509 markers were implemented in the subsequent genome-wide analysis.

### Feed efficiency and feeding behaviour traits

Raw feed data contained records from each entry to the feeding machine during the test period. Errors in single visit feed intake records were identified following the algorithm developed by Casey et al. (2005) and removed from the data. The first week of the test was removed from further analysis to allow pigs an adaptation period to the feed dispenser. Average daily feed intake (DFI, g/day), average daily occupation time in the electronic feeder (DOT, min/day), average daily number of visits to the electronic feeder (DFV, count), and feeding rate (DFR, g/min/day) were calculated over the test period for each animal. DOT was calculated as the sum of times an animal spent at the feeder divided by the days of feeding records. DFR represents the ratio between DFI and DOT. FCR during the test period was expressed by the quotient of individual DFI and body weight gain (difference between end and start body weight). Prior to association analyses, DFV was transformed using the square root function $[\sqrt {({\text{DFV}} + 1)} ]$ to fit a normal distribution. The descriptive statistics are given in Table 2.

**Table 2:** Descriptive statistics of feed efficiency and feeding behaviour traits analysed in the Maxgro population

Trait	Abbreviation	*n*	Mean	SD	Min	Max
Feed conversion ratio (g/g)	FCR	823	2.26	0.23	1.38	3.57
Daily feed intake (g/day)	DFI	843	2733.4	320.0	1488	3924
Daily feeder visits (count/day)	DFV	843	4.29	0.90	2.65	8.72
Daily occupation time (min/day)	DOT	843	61.95	11.37	32	99
Daily feeding rate (g/min/day)	DFR	843	45.38	8.79	24	79

### Genome-wide association analysis (GWAS)

For the identification of QTL, an integrated strategy, combining both single-marker and multi-marker approaches, was applied to the dataset as previously described (Reyer et al. 2015). In brief, mixed linear models were carried out for each trait using JMP genomics 6 (SAS Institute, Cary, USA). These included random effects of sire line and dam line to account for relatedness between boars. Furthermore, the average animal age during the test period was considered as covariate in the models accounting for age-related differences in feeding behaviours as well as age-related differences in body weight. SimpleM was used to assess the effective number of independent tests (*n* = 22,811) based on the imputed genotype matrix (Gao et al. 2008). Accordingly, significance thresholds were set to *P* = 4.38*E*−05 [−log(*p* value) = 4.36] for suggestive significance and *P* = 2.19*E*−06 [−log(*p* value) = 5.66] for genome-wide significance. Least square means were extracted for each genotype class and the explained phenotypic variance of each SNP was deduced from the squared multiple correlation of the regression obtained from the mixed model analysis. Based on the information of markers used for the genome-wide analyses, linkage disequilibrium (LD) (*r* ^2^) between SNPs was analysed for each chromosome employing the Haploview software (v4.2) as previously described (Barrett et al. 2005; Reyer et al. 2015). Linkage blocks were defined using the ‘solid spine of LD’ algorithm provided by Haploview.

The applied multi-marker method integrated the information of all SNPs located within 2577 consecutive windows of 1 Mb (without UWGS). Based on the chromosome-wide results of LD analyses, the average LD between markers at a distance of 1 Mb was 0.12 ± 0.03 (mean ± SD). Moreover, 1-Mb windows comprised on average 20 SNP markers. Analyses were performed using a Bayesian approach implemented in the GenSel programme available via the CyVerse discovery environment (http://cyverse.org) (Fernando and Garrick 2008). Parameters were set to process 51,000 iterations, with the first 1000 cycles discarded as burn-in and an output frequency of 50. The fraction of SNPs having zero effects was set to *π* = 0.995. Hence, on average, 260 SNPs contributing to the genetic variance were fitted per iteration. Finally, results were summarised within 1-Mb windows and estimates of the genetic variance explained by each window were extracted. In total, 2577 1-Mb windows were retrieved which were assumed to have a theoretical proportion to the genetic variance of about 0.04% (100%/2577 windows). Windows having contributions to the genetic variance that were more than ten times higher than this theoretical contribution (explained genetic variance >0.5%) were considered in subsequent analyses. For these 1-Mb windows, information obtained from single-marker analyses was used to calculate the 95% confidence intervals (CI) according to the quick method proposed by Li (2011). Genomic windows were screened for functional candidate genes combining the information of the pig genome resource (http://www.ensembl.org/, release 86, accessed October 2016) and of functional gene annotations implemented in the GeneCards database (http://www.genecards.org/, accessed October 2016).

## Results

### Feed conversion ratio (FCR)

**Fig. 1:**
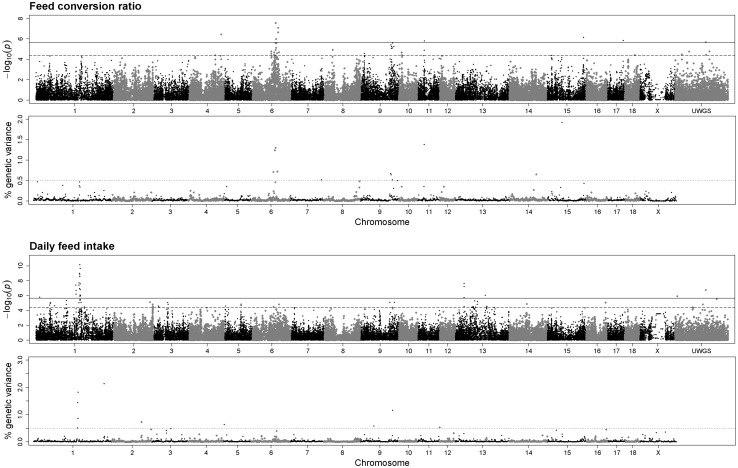
Manhattan plots indicating QTL for feed efficiency traits in a terminal sire line population. Results from single-marker GWAS (*upper plot*) and a multi-marker approach (*lower plot*) are depicted for feed conversion ratio and daily feed intake, respectively. *Bold* and *dashed lines* indicate the threshold for genome-wide [−log(*p* value) = 5.66] and suggestive significance [−log(*p* value) = 4.36] of association. *Dotted lines* represent contributions of a 1-Mb genomic window to the additive genetic variance of the traits above 0.5%

The genome-wide analyses of FCR revealed 12 1-Mb windows on six different *S. scrofa* chromosomes (SSC) contributing more than 0.5% to the genetic variance of the trait (Table 3). The most prominent region, revealed by single- and multi-marker analyses, was located between 88.0 and 107.7 Mb on SSC 6 and includes 16 significantly associated SNPs (*P* ≤ 4.38*E*−05) and four 1-Mb windows exceeding the threshold level (Fig. 1). The highest significantly associated SNPs in each 1-Mb window explained between 2.6 and 4.2% of the phenotypic variance (Online Resource 1). A potential candidate gene is *MACF1*, which is a large gene mapping in the window between 88.0 and 89.0 Mb. The indicated LD block in this region was defined by ALGA0036014 (87.6 Mb) and ALGA0109191 (88.5 Mb) (Online Resource 2). A second candidate gene deduced for SSC6 was *AQP4* at 104.3 Mb which is located in a 0.7 Mb spanning LD block from ASGA0095497 (104.1 Mb) to ALGA0111332 (104.8 Mb). SSC 9 harbours another wide QTL region comprising the genome section between 120.0 and 128.0 Mb in which three 1-Mb windows showed contribution to the genetic variance of FCR above 0.5%. Linkage analysis revealed 32 LD blocks in this genomic region with the largest block comprising markers between 122.9 and 123.5 Mb. The genetic window located between 25.0 and 26.0 Mb on SSC 11 explained 1.38% of the genetic variance of FCR. In addition, two SNPs (at 24.6 and 25.1 Mb) pointed to this QTL. Thereby, SNP ASGA0050399 located at 24.6 Mb mapped in an intronic region of *DnaJC15*. The highest contribution to the genetic variance of FCR revealed by Bayesian analysis was assigned to a region between 57.3 and 57.9 Mb on SSC 15. The window explained 1.92% of the genetic variance. However, the highest associated single-marker (ALGA0085398) was located at 57.81 Mb and did not exceed the significance threshold (*P* = 6.8*E*−05). Homozygous carriers of the major allele of ALGA0085398 showed an improvement in FCR by 0.12 g/g compared to homozygous carriers of the minor allele. Other 1-Mb genomic windows exclusively supported by multi-marker analyses are summarized in Table 3.

**Table 3:** Genomic 1-Mb windows contributing to feed efficiency and feeding behaviour traits obtained from the integration of single- and multi-marker genome-wide association analyses in a terminal boar population (*n* = 846)

Trait	SSC^a^	1-Mb window (Mb)	% Var^b^	No. SNP^c^	SNP [−log_10_(*p* value)]^d^	Putative candidate genes (position)
FCR	6	88–89	0.71	4	ALGA0036056 (4.68)	*MACF1* (88.0–88.4 Mb), *MFSD2A* (88.9 Mb)
	6	94–95	1.24	3	ALGA0122144 (7.55)	*EPB41L3* (94.7–94.8 Mb)
	6	97–98	1.30	1	MARC0089589 (5.59)	*MYOM1* (96.5–96.6 Mb)
	6	104–105	0.72	2	ALGA0115465 (7.08)	*AQP4* (104.4 Mb)
	7	124–125	0.52	0	ALGA0045316 (4.12)	*GLRX5* (124.0 Mb), *TCL1B* (124.1 Mb)
	9	120–121	0.67	1	H3GA0053804 (5.42)	*ENSSSCG00000022338* (120.7–120.9 Mb)
	9	122–123	0.64	1	MARC0083358 (5.07)	–
	9	127–128	0.52	2	ALGA0054777 (5.59)	*TNFSF4* (127.0 Mb), *TNFSF18* (127.1–127.2 Mb)
	9	148–149	0.50	0	ALGA0105115 (2.50)	*PLXNA2* (148.1–148.3 Mb)
	11	25–26	1.38	1	H3GA0031644 (5.81)	*ENOX1* (24.2–24.5 Mb), *DNAJC15* (24.6–24.7 Mb), *TNFSF11* (24.1–24.2 Mb)
	14	107–108	0.65	0	ALGA0080254 (2.68)	*ENSSSCG00000029076* (127.5–107.7 Mb)
	15	57–58	1.92	0	ALGA0085398 (4.17)	*UNCSD* (57.4–57.6 Mb)
DFI	1	176–177	0.51	2	ASGA0004976 (8.99)	*TNFRSF11A* (176.6–176.7 Mb), *PIGN* (176.8–176.9 Mb)
	1	177–178	1.44	6	ALGA0006621 (10.15)	*RNF152* (177.1 Mb)
	1	178–179	0.86	4	INRA0004955 (10.15)	*MC4R* (178.6 Mb)
	1	179–180	1.82	8	MARC0013872 (9.66)	*LMAN1* (179.2 Mb), *ENSSSCG00000004911* (179.3–179.4 Mb)
	1	283–284	2.13	0	ALGA0009308 (3.71)	*SUSD1* (283.6–283.7 Mb), *ENSSSCG00000022780* (283.4–283.5 Mb)
	2	118–119	0.73	0	H3GA0007369 (3.45)	*ENSSSCG00000014192* (118.3 Mb)
	5	2–3	0.63	1	ALGA0029934 (4.59)	*PARVB* (2.1–2.2 Mb), EFCAB6 (2.6–2.8 Mb)
	9	53–54	0.58	0	MARC0025903 (4.16)	*ENSSSCG00000026007* (53.7–53.8 Mb), *SC5D* (53.6 Mb)
	9	128–129	1.16	1	ALGA0054797 (4.41)	*ENSSSCG00000026540* (128.2–128.3 Mb), *ENSSSCG00000022119* (128.5–128.6 Mb)
	12	0–1	0.53	0	ALGA0116599 (3.48)	*NARF* (0.1 Mb)
DOT	1	176–177	3.64	7	INRA0004895 (12.58)	*TNFRSF11A* (176.6–176.7 Mb), *PIGN* (176.8–176.9 Mb)
	1	177–178	0.89	10	ASGA0004992 (11.07)	*RNF152* (177.1 Mb)
	1	178–179	2.55	1	ALGA0006623 (11.11)	*MC4R* (178.6 Mb)
	1	179–180	6.64	10	INRA0004984 (13.28)	*LMAN1* (179.2 Mb), *ENSSSCG00000004911* (179.3–179.4 Mb)
	4	102–103	0.87	1	H3GA0013527 (5.49)	*IQGAP3* (102.1–102.2 Mb)
	7	127–128	0.55	4	MARC0012014 (4.95)	–
	8	141–142	0.99	1	ALGA0049934 (5.10)	*SLC10A6* (141.3 Mb), *PTPN13* (141.4–141.6 Mb)
	9	23–24	1.09	2	ASGA0042072 (4.99)	*CTSC* (24.1 Mb)
	13	12–13	0.63	0	MARC0091244 (1.76)	*NR1D2* (12.2 Mb), *THRB* (12.4–12.5 Mb)
DFV	1	303–304	0.70	0	ASGA0007897 (2.57)	*CRAT* (303.4 Mb)
	6	105–106	0.67	5	ALGA0103394 (6.39)	–
	7	2–3	0.85	1	MARC0035078 (4.58)	*SLC22A23* (2.1–2.2 Mb), *PXDC1* (2.3 Mb), *ECI2* (2.5 Mb)
	14	50–51	0.66	0	H3GA0040087 (3.52)	*MTMR3* (49.9–50.1 Mb), *MORC2* (50.9 Mb), *USMG5* (50.5–50.6 Mb)
	16	8–9	0.51	1	ALGA0112899 (5.71)	*CDH18* (8.7–8.9 Mb)
DFR	4	102–103	3.19	1	H3GA0013527 (5.95)	*IQGAP3* (102.1–102.2 Mb)
	7	127–128	0.81	0	H3GA0023563 (4.06)	–
	8	128–129	1.09	6	ASGA0039774 (7.20)	*PPP3CA* (*ENSSSCG00000009172*; 128.5–128.8 Mb), *ENSSSCG00000022835* (128.9 Mb)
	14	130–131	0.71	0	ALGA0081429 (4.05)	–
	17	26–27	0.90	1	MARC0085963 (5.71)	–
	18	50–51	0.86	0	MARC0055314 (3.00)	*ENSSSCG00000016708* (50.3–50.4 Mb)
	X	109–110	0.52	3	H3GA0055497 (6.07)	*HTR2C* (108.6–108.9 Mb), *PLS3* (109.5 Mb)
	X	110–111	0.57	1	H3GA0051891 (5.01)	*SLC6A14* (110.1 Mb)

*FCR* feed conversion ratio, *DFI* daily feed intake, *DOT* daily occupation time, *DFV* daily feeder visit, *DFR* daily feeding rate

^a^ *Sus scrofa* chromosome according to genome build 10.2

^b^Genetic variance explained by the 1-Mb window in percent

^c^Number of significantly associated SNPs [−log(*p* value) ≥4.36] in the corresponding 1-Mb window obtained from single-marker analysis

^d^Single-nucleotide polymorphism (SNP) that showed the highest significant association according to single-marker analysis

### Daily feed intake (DFI)

The integration of both genome-wide approaches revealed ten 1-Mb windows of which five are located on SSC 1 (Table 3). Specifically, a strong QTL region on SSC1 mapped between 174.4 and 183.3 Mb covering the *MC4R* locus (~178.6 Mb). In this region 27 SNPs were significantly associated with DFI (Fig. 1). In addition, four consecutive 1-Mb windows from 176.0 to 180.0 Mb explain in total 4.63% of the additive genetic variance of DFI. The connection between these 1-Mb windows is supported by an average LD between adjacent markers of 0.42 (average distance between markers is 83 kb) and by common LD blocks (Online Resource 2). Two other QTL were synergistically identified by both GWAS methods located at 2.0–2.9 Mb and 128.0–129.0 MB on SSC5 and 9, respectively. Uniquely, single-marker analysis revealed a QTL indicated by a cluster of three neighbouring SNPs mapping at approximately 34.5 Mb on SSC 13 (Fig. 1). All three markers showed genome-wide-significant association with DFI. Two of these SNPs are located beside and in the *PFKFB4* gene. The effects of the highest significantly associated SNPs in each 1-Mb window on DFI are given in Online Resource 1.

### Daily occupation time (DOT)

**Fig. 2:**
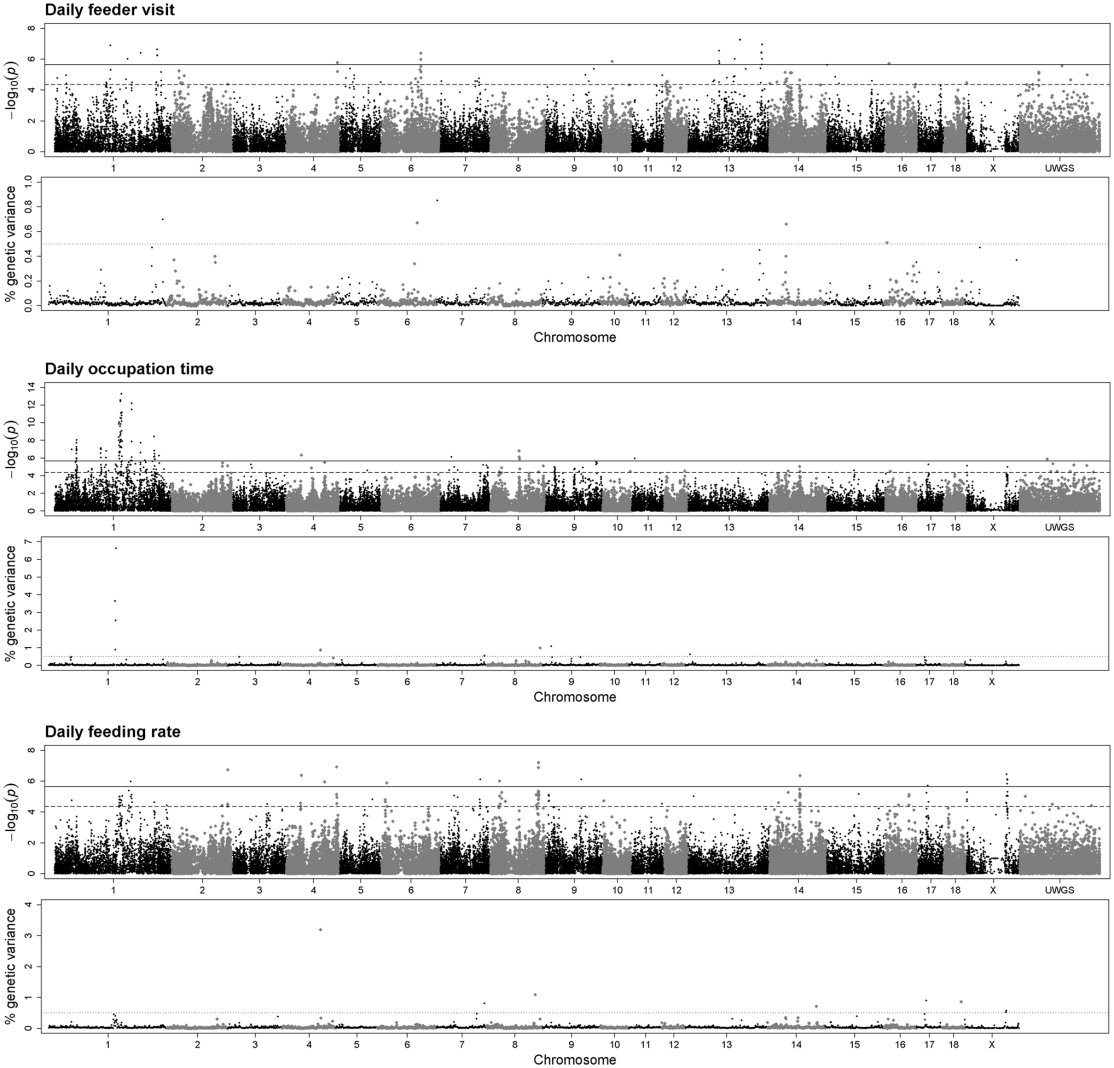
Illustration of the results obtained from single-marker (*upper plot*) and multi-marker (*lower plot*) GWAS for three different feeding behaviour traits in pigs. *Bold* and *dashed lines* represent the threshold for genome-wide significance [−log(*p* value) = 5.66] and suggestive significance [−log(*p* value) = 4.36] applied for single-marker analysis. *Dotted lines* indicate for 1-Mb genomic regions which contribute more than 0.5% to the additive genetic variance

In accordance with the analyses of DFI, the genomic section between 172.0 and 181.0 Mb on SSC1 was the most conspicuous region in single-marker analysis of DOT (Fig. 2). In this region, 59 markers were significantly associated with DOT, of which 45 SNPs exceed the threshold of genome-wide significance (*P* ≤ 2.19*E*−06). According to DFI, the same four 1-Mb windows between 176.0 and 179.0 Mb on SSC 1 were obtained in the multi-marker analysis. The estimated contribution of the whole 4-Mb region to the genetic variance of DOT was 13.72%. The highest significantly associated SNP in this QTL (INRA0004984) affected a shift in DOT from 64.8 to 56.3 min/day (Online Resource 1). Other QTL with contributions to the genetic variance in DOT were identified on SSC 4, 7, 8, 9, and 13, as summarized in Table 3. Furthermore, single-marker analysis uncovered several significantly associated SNPs located on SSC 1 indicating for genetic contributions of these genomic regions to the individual variation in the time an animal spent at the feeder (e.g. at 55.9–60.5 Mb and at 260.7–272.0 Mb).

### Daily feeder visit (DFV)

Multi-marker analyses revealed five 1-Mb windows with contributions to the genetic variance in DFV between 0.51 and 0.85% (Table 3). The 1-Mb window on SSC 6 between 105.0 and 106.0 Mb explained a proportion of 0.67% of the genetic variance in DFV and was further indicated by five significantly associated SNPs. Another obtained QTL, indicated by both approaches, covered the region between 2.0 and 2.9 Mb on SSC 7 (Fig. 2). In this window, SNP MARC0035078 exceeded the threshold of significant association in single-marker analysis (*P* = 2.63*E*−05) and the 95% CI pointed to *ECI2* as putative candidate gene (Online Resource 2). The 1-Mb window on SSC 16 (8.0–8.8 Mb) and the corresponding 95% CI (Online Resource 2) highlighted *CDH18* as positional candidate gene. It is further supported by the significant association of SNP ALGA0112899 mapping in the intronic region of this gene.

### Daily feeding rate (DFR)

For DFR, eight 1-Mb windows were obtained (Table 3). Two of them completely overlap with regions also identified in the analyses of DOT. Specifically, the window between 102.1 and 102.9 Mb on SSC 4 showed the highest contribution to the genetic variance in DFR (3.19%). Screening for candidates, by utilizing information of LD and CI deduced from single-marker analysis, revealed *IQGAP3* located at approximately 102.6 Mb as positional candidate gene (Online Resource 2). The second overlapping window between both traits covered the region from 127.0 to 128.0 Mb on SSC 7. Single-marker analysis further revealed a putative QTL between 125.0 and 130.1 Mb on SSC 8 (Fig. 2). In this region, 10 SNPs showed significant association with DFR, of which four mapped in the *PPP3CA* gene. In addition, the 1-Mb window (128.0–129.0 Mb) containing the *PPP3CA* locus explained a proportion of 1.09% to the genetic variance in DFR. Regarding SSC X, two adjacent windows between 109.0 and 110.9 Mb showed contribution to the genetic variance above the threshold of 0.5%. LD analyses revealed a common QTL comprising both 1-Mb windows with a contribution to the genetic variance in DFR of 1.09%. The QTL was also indicated by significantly associated SNPs obtained from single-marker analysis and harboured putative functional and positional candidates, namely *PLS3* and *SLC6A14* (Online Resource 2).

## Discussion

The conducted genome-wide association study revealed 44 1-Mb windows contributing to the five analysed FE and feeding behaviour traits. Analyses of FCR and DFI revealed 12 and 10 QTL regions, respectively. For DOT, DFV, and DFR, as feeding behaviour traits, 9, 5, and 8 prominent genomic regions were identified. Completely overlapping 1-Mb regions were found on SSC 1 for DOT and DFI as well as on SSC 4 and SSC 7 for DOT and DFR. As exemplified by the highest significant associated SNP in the QTL on SSC 7 (H3GA0013527), the allele substitution leads to a reduction in DOT and an increase in DFR. These results provide evidence for a common genetic foundation of the analysed traits and, moreover, for a genetic basis of certain feeding strategies as already indicated by comparing feeding behaviours of different pig breeds (Fernández et al. 2011).

Some of the identified QTL regions partially overlap with the previous studies. Specifically, QTL for FCR on SSC 11 and 14 and for DFR on SSC 7 were consistently identified in a Meishan × Large White cross using microsatellite-based analysis (Houston et al. 2005). In addition, Gilbert et al. (2010) reported marginally significant QTL for FCR on SSC 6 (at 125 cM) and SSC 15 (at 51 cM) spanning herein identified 1-Mb windows (Gilbert et al. 2010). For DFI, the designated genomic region on SSC 2 was also previously indicated by microsatellite analysis in an F2 population of pigs originating from a cross of Pietrain and a commercial dam line (Duthie et al. 2008; Shirali et al. 2013). Shirali et al. (2013) showed that this QTL region on SSC 2 is also influenced by residual energy intake as partial measure of feed efficiency. The same study revealed overlapping QTL for FCR on SSC 6 (region from 104 to 105 Mb), for FCR and residual energy intake on SSC 7 (region from 124 to 125 Mb), and for DFI as well as protein and lipid deposition in the body on SSC 9 (region from 128 to 129 Mb). Moreover, the FCR-associated genomic region on SSC 1 (region from 283 to 284 Mb) overlaps with a QTL for average daily gain and protein deposition in the body. Compared to the previous GWAS using high-density SNP arrays in other pig breeds with focus on FCR (Sahana et al. 2013; Wang et al. 2015) and feeding behaviour (Guo et al. 2015), no exact overlapping regions were observed. However, comparisons of pig breeds regarding feeding behaviour and FE traits revealed breed-specific differences in feeding strategies and genetic contributions especially for feeding behaviour traits (Fernández et al. 2011; Do et al. 2013).

The obtained QTL for DFI and DOT located on SSC 1 pointed to the *MC4R* locus at 178.5 Mb which is widely discussed for contributions to the phenotypic variance of feeding behaviour traits and feed intake in pigs (Kim et al. 2000). The proposed causal *MC4R* (Asp298Asn) mutation affects energy homeostasis influencing back fat thickness, weight gain, and feed intake but at the same time showing inconsistencies in its penetrance in different populations (Kim et al. 2000; Piórkowska et al. 2010; Dvořáková et al. 2011). Moreover, the current and other studies provided evidence that the genomic region around *MC4R* includes other, potentially pleiotropic-acting, genetic variants influencing FE and feeding behaviour traits (Jiao et al. 2014). The most interesting positional and functional candidate genes revealed by the combined GWAS approach comprise the Aquaporin 4 encoding *AQP4*, the alpha isoform of the catalytic subunit of calcineurin encoded by *PPP3CA*, the IQ Motif Containing GTPase Activating Protein 3 gene (*IQGAP3*), and *DnaJC15* which encodes for a protein belonging to the DnaJC subfamily of co-chaperones.

Aquaporins are water-selective channels embedded in the cell membrane. As such, they influence the water permeability, thus, regulating the cellular water balance. Beside the abundance of *AQP4* in cells of the central nervous systems, the gene is also expressed in skeletal muscle fibres where the gene product assists in the osmotic-driven transport of water from blood to muscle cells (Frigeri et al. 2004). Moreover, *AQP4* expression levels in muscle cell membranes are increased by endurance exercises to regulate metabolic needs during physical activity (Wakayama 2010; Basco et al. 2013). Aquaporins represents an interesting class of molecules in the context of FE as the cellular and organismal incorporation of water could considerably affect weight gain contributing to FCR.

Calcineurin is activated by increased intracellular calcium levels and initiates tissue-specific effects controlling immune system functions, body weight, and energy homeostasis. Specifically, a mouse model with an impaired but functional calcineurin protein showed decreased plasma concentrations of molecules central to the regulation of appetite, satiety, and energy expenditure like leptin, adiponectin, and free fatty acids (Pfluger et al. 2015). Feed intake and FE values of these mice differ from wild-type controls. Accordingly, the evidence for a genetic contribution of the *PPP3CA* locus to the variation in feeding behaviour traits is in agreement with the role of calcineurin in the sensation and regulation of energy status (Wang et al. 2012; De Andrade et al. 2015; Pfluger et al. 2015). Ultimately, the reactivity of calcium-mediated signalling cascades via calcineurin and subsequent regulations of appetite and satiety could potentially influence individual feeding rates of pigs with putative implications on growth rate traits, as previously suggested (Wang et al. 2015). One of the molecule families involved in the downstream signalling induced by calcium or calcineurin is IQGAPs (Smith et al. 2015). Thus, the GWAS-derived evidence for an association of *IQGAP3* with DOT and DFR ties in with putative effects on calcium/calcineurin-mediated signalling pathways contributing to the variation of feeding rate and time spent for eating. Moreover, functional annotations of IQGAP3 revealed participation of the protein in cell proliferation, cytoskeletal dynamics, cell–cell adhesion, and intracellular signalling (Nojima et al. 2008; Hedman et al. 2015).

DnaJC15 acts as a negative regulator of the mitochondrial respiratory chain by controlling complex I activity. Thus, it is involved in the cellular energy production which is suggested to be highly relevant for explaining individual variations in FE traits in livestock species (Bottje and Carstens 2009). Specifically, the absence of DnaJC15 protein increases mitochondrial complex I activity and induces ATP production (Hatle et al. 2013). Consequently, knock-out animal models showed enhanced hepatic lipid metabolism affecting the accumulation of body lipids. However, the increase in hepatic turnover results in a rapid fat loss after fasting with lower levels of free fatty acids, and an increased liver glycogenesis. Accordingly, *DnaJC15*, located in the designated genomic region on SSC11, is a promising functional candidate gene for FCR by affecting the efficiency of ATP production and the utilization of metabolic routes.

Moderate genetic correlations between and among FE and feeding behaviour traits illustrate that animal selection on FE measures (either residual feed intake or FCR) affects the genetics of feeding behaviours (Do et al. 2013; Shirali et al. 2015). Moreover, both trait categories are moderately linked to production traits like back fat deposition and lean percentage from a genetic point of view. Whilst FE has been considered in pig breeding for decades, the implementation of feeding behaviour traits in breeding programmes is not established. This, of course, is based on the fact that the revealed relationships between feeding behaviour traits are inconsistent among breeds and that the physiological and economic consequences of distinct feeding strategies are still unclear (Fernández et al. 2011; Do et al. 2013). Nevertheless, the detailed genetic and phenotypic evaluation of grower/finisher pigs regarding their feeding behaviour can provide biomarkers to assess and predict implications on animal health and welfare. Specifically, DFV and DOT could be used as valuable indicators for monitoring pig management and social interaction among group-housed pigs (Hoy et al. 2012; Brown-Brandl et al. 2013). The breeder’s selection regarding DFR could further influence the function and integrity of the digestive system, e.g., in the etiology of gastric ulcers (Swaby and Gregory 2012). However, further work is needed to clarify the physiological consequences of feeding behaviours, for instance, on stomach and gut health. Therefore, the identification and investigation of candidate genes for feeding behaviour traits and their relation of FE traits will provide considerable insights in underlying molecular mechanisms and pathways.

## Conclusion

The genome-wide association analyses of the terminal sire line population revealed major QTL for feeding behaviour traits including DOT, DFV, and DFR on SSCs 1, 4, 6, 7, 8, and 14. For FCR and DFI as FE traits, prominent genomic regions were identified on SSCs 1, 6, 9, and 11. These regions contain several candidate genes with regard to their positional and functional evidence for an association with FE and feeding behaviours. Functional annotations of these genes imply that although established pig breeds are already improved regarding their resource allocation and efficiency, the genetics contributing to cellular ATP generation, water homeostasis, and energy metabolism play a considerable role and are highly relevant for the variation of analysed traits. Nevertheless, an essential next step to verify the contributions of identified genomic region to the traits will be to validate the robustness of their association in independent pig populations. Moreover, the dissection of QTL regions will provide additional information of putative genetic factors involved in both groups of traits. Further investigation is also needed to clarify the connection between distinct feeding behaviour patterns and performance, health, and welfare traits to consider their implementation in breeding programmes and pig husbandry.

## Electronic supplementary material

Below is the link to the electronic supplementary material.

Supplementary material 1 (PDF 11 kb)

Supplementary material 2 (PDF 25 kb)
