# Supplementary material for: Exploring the genetics of feed efficiency and feeding behaviour traits in a pig line highly selected for performance characteristics
Source: Mol Genet Genomics. 2017 May 12;292(5):1001–11. doi: 10.1007/s00438-017-1325-1 (PMC5594041; doi:10.1007/s00438-017-1325-1)
Supplement: Supplementary file 1 — Supplementary material 1 (PDF 11 kb) [file 438_2017_1325_MOESM1_ESM.pdf]

Exploring the genetics of feed efficiency and feeding behaviour traits in a pig line highly selected for performance characteristics

Molecular Genetics & Genomics

Henry Reyer, Mahmoud Shirali, Siriluck Ponsuksili, Eduard Murani, Patrick F. Varley, Just Jensen, Klaus Wimmers*

*Corresponding author:

Klaus Wimmers
Leibniz Institute for Farm Animal Biology
Wilhelm-Stahl-Allee 2
18196 Dummerstorf, Germany
Email: wimmers@fbn-dummerstorf.de

Supplementary Table 1. Phenotypic effects of markers which showed the highest significant association with feed efficiency and feeding behaviour traits in the identified 1-Mb regions.

Trait[1]	Marker	Chr.	Position	Major allele (0)	Minor allele (2)	MAF[2]	LSM (SE)[3] 0	LSM (SE)[3] 1	LSM (SE)[3] 2	$-\log_{10}$ (P-value)	%Var[4]
**FCR**	ALGA0036056	6	88197331	C	T	0.298	2.24 (0.02)	2.30 (0.02)	2.35 (0.03)	4.68	2.6
**FCR**	ALGA0122144	6	94464352	G	A	0.472	2.33 (0.02)	2.28 (0.02)	2.20 (0.03)	7.55	4.2
**FCR**	MARC0089589	6	97199299	G	A	0.330	2.24 (0.02)	2.29 (0.02)	2.37 (0.03)	5.59	3.1
**FCR**	ALGA0115465	6	104376158	G	T	0.368	2.24 (0.02)	2.29 (0.02)	2.38 (0.03)	7.08	3.9
**FCR**	ALGA0045316	7	124385985	T	G	0.352	2.24 (0.02)	2.29 (0.02)	2.35 (0.03)	4.12	2.3
**FCR**	H3GA0053804	9	120749130	G	A	0.417	2.24 (0.02)	2.27 (0.02)	2.35 (0.03)	5.42	3.0
**FCR**	MARC0083358	9	122944920	C	T	0.181	2.25 (0.02)	2.33 (0.02)	2.34 (0.05)	5.07	2.8
**FCR**	ALGA0054777	9	127321307	A	G	0.498	2.22 (0.02)	2.28 (0.02)	2.33 (0.02)	5.59	3.1
**FCR**	ALGA0105115	9	148629187	A	G	0.292	2.31 (0.02)	2.26 (0.02)	2.25 (0.03)	2.50	1.4
**FCR**	H3GA0031644	11	25174659	A	G	0.180	2.24 (0.02)	2.33 (0.02)	2.35 (0.05)	5.81	3.2
**FCR**	ALGA0080254	14	107547829	T	C	0.372	2.25 (0.02)	2.29 (0.02)	2.33 (0.03)	2.68	1.5
**FCR**	ALGA0085398	15	57805024	A	C	0.338	2.25 (0.02)	2.28 (0.02)	2.37 (0.03)	4.17	2.3
**DFI**	ASGA0004976	1	176492950	T	C	0.096	2738 (34)	2561 (41)	2618 (110)	8.99	4.8
**DFI**	ALGA0006621	1	177818653	G	A	0.154	2755 (34)	2599 (37)	2512 (76)	10.15	5.4
**DFI**	INRA0004955	1	178254757	T	C	0.154	2755 (34)	2599 (37)	2512 (76)	10.15	5.4
**DFI**	MARC0013872	1	179327620	T	G	0.137	2751 (34)	2593 (38)	2546 (76)	9.66	5.2
**DFI**	ALGA0009308	1	283682083	T	C	0.405	2652 (36)	2731 (35)	2772 (42)	3.71	2.0
**DFI**	H3GA0007369	2	118010721	C	T	0.316	2693 (34)	2699 (34)	2838 (46)	3.45	1.9
**DFI**	ALGA0029934	5	2658163	A	G	0.213	2748 (34)	2660 (35)	2576 (63)	4.59	2.5
**DFI**	MARC0025903	9	53697784	T	C	0.327	2749 (34)	2714 (34)	2592 (42)	4.16	2.3
**DFI**	ALGA0054797	9	128255464	T	C	0.323	2670 (34)	2742 (35)	2815 (46)	4.41	2.4
**DFI**	ALGA0116599	12	103340	G	A	0.418	2750 (35)	2692 (34)	2628 (40)	3.48	1.9
**DOT**	INRA0004895	1	176231881	G	A	0.313	63.8 (1.4)	59.6 (1.4)	54.7 (1.7)	12.58	6.7
**DOT**	ASGA0004992	1	177744561	A	G	0.414	64.7 (1.4)	59.9 (1.4)	57.3 (1.6)	11.07	5.9
**DOT**	ALGA0006623	1	178024855	T	C	0.420	64.7 (1.4)	60.1 (1.4)	57.1 (1.6)	11.11	5.9
**DOT**	INRA0004984	1	179188746	T	C	0.400	64.8 (1.4)	60.0 (1.4)	56.3 (1.6)	13.28	7.0
**DOT**	H3GA0013527	4	102162642	T	C	0.368	63.1 (1.4)	59.7 (1.3)	58.0 (1.6)	5.49	3.0
**DOT**	MARC0012014	7	127278151	C	T	0.340	60.2 (1.2)	60.4 (1.2)	66.0 (1.6)	4.95	2.7
**DOT**	ALGA0049934	8	141678271	A	G	0.472	58.8 (1.3)	61.2 (1.2)	64.2 (1.4)	5.10	2.8
**DOT**	ASGA0042072	9	23437153	T	C	0.360	58.9 (1.5)	61.9 (1.4)	64. (1.7)	4.99	2.7
**DOT**	MARC0091244	13	12500465	T	C	0.449	59.4 (1.3)	60.8 (1.2)	62.8 (1.4)	1.76	1.0
**DFV**	ASGA0007897	1	303622361	T	C	0.379	2.33 (0.02)	2.30 (0.02)	2.26 (0.03)	2.57	1.4
**DFV**	ALGA0103394	6	105613840	C	T	0.335	2.34 (0.02)	2.27 (0.02)	2.26 (0.03)	6.39	3.4
**DFV**	MARC0035078	7	2509192	T	C	0.301	2.33 (0.02)	2.28 (0.02)	2.25 (0.03)	4.58	2.5
**DFV**	H3GA0040087	14	50090206	A	T	0.476	2.35 (0.02)	2.30 (0.02)	2.28 (0.02)	3.52	1.9
**DFV**	ALGA0112899	16	8752527	T	C	0.398	2.26 (0.02)	2.32 (0.02)	2.36 (0.03)	5.71	3.1
**DFR**	H3GA0013527	4	102162642	T	C	0.368	43.8 (0.8)	46.6 (0.7)	47.9 (1.0)	5.95	3.2
**DFR**	H3GA0023563	7	127537686	G	T	0.499	45.6 (0.9)	46.8 (0.8)	43.7 (0.9)	4.06	2.2
**DFR**	ASGA0039774	8	128899782	T	C	0.395	44.3 (0.8)	45.6 (0.8)	49.2 (1.0)	7.20	3.9
**DFR**	ALGA0081429	14	130733740	A	C	0.371	47.3 (0.9)	44.8 (0.8)	44.4 (1.1)	4.05	2.2
**DFR**	MARC0085963	17	26443872	C	T	0.392	47.1 (0.8)	45.4 (0.8)	42.3 (1.1)	5.71	3.1
**DFR**	MARC0055314	18	50389754	T	C	0.171	46.5 (0.8)	44.2 (0.9)	44.1 (1.9)	3.00	1.6
**DFR**	H3GA0055497	X	109562447	G	A	0.293	44.7 (0.6)	-	47.9 (0.7)	6.07	2.8
**DFR**	H3GA0051891	X	110186817	G	A	0.462	46.9 (0.7)	-	44.2 (0.8)	5.01	2.3

[1] FCR – feed conversion ratio (g/g), DFI – daily feed intake (g/d), DOT – daily occupation time (min/d), DFV – daily feeder visit (count/d), DFR – daily feeding rate (g/min/d)

[2] MAF – minor allele frequency

[3] Least square means (LSM) and standard errors of the LSM are presented for the numeric coded genotypes (0 – homozygous for the major allele, 1 – heterozygous, 2 – homozygous for the minor allele)

[4] Phenotypic variance in percent explained by the SNP
