# Supplementary material for: Exploring the genetics of feed efficiency and feeding behaviour traits in a pig line highly selected for performance characteristics
Source: Mol Genet Genomics. 2017 May 12;292(5):1001–11. doi: 10.1007/s00438-017-1325-1 (PMC5594041; doi:10.1007/s00438-017-1325-1)
Supplement: Supplementary file 2 — Supplementary material 2 (PDF 25 kb) [file 438_2017_1325_MOESM2_ESM.pdf]

Exploring the genetics of feed efficiency and feeding behaviour traits in a pig line highly selected for performance characteristics

Molecular Genetics & Genomics

Henry Reyer, Mahmoud Shirali, Siriluck Ponsuksili, Eduard Murani, Patrick F. Varley, Just Jensen, Klaus Wimmers*

*Corresponding author:

Klaus Wimmers
Leibniz Institute for Farm Animal Biology
Wilhelm-Stahl-Allee 2
18196 Dummerstorf, Germany
Email: wimmers@fbn-dummerstorf.de

Supplementary Table 2. Linkage disequilibrium blocks and confidence intervals derived from markers which showed the highest significant association with feed efficiency and feeding behaviour traits in the identified 1-Mb regions.

Trait[1]	SNP (-log$_{10}$[p-value])[2]	SSC[3]	SNP position in bp	1-Mb window (Mb)	LD block limits (region in Mb)[4]	95% confidence interval (CI) in bp[5]
**FCR**	ALGA0036056 (4.68)	6	88197331	88 - 89	ALGA0036014 : ALGA0109191 (87.62 - 89.50)	88054214 - 88384740
	ALGA0122144 (7.55)	6	94464352	94 - 95	MARC0096269 : MARC0034518 (94.38 - 94.78)	94462469 - 94465549
	MARC0089589 (5.59)	6	97199299	97 - 98	ASGA0105222 : MARC0089589 (96.76 - 97.20)	97199299 - 97205162
	ALGA0115465 (7.08)	6	104376158	104 - 105	ASGA0095497 : ALGA0111332 (104.10 - 104.81)	104367172 - 104380166
	ALGA0045316 (4.12)	7	124385985	124 - 125	ASGA0036707 : ALGA0045316 (124.36 - 124.39)	124380264 - 124391232
	H3GA0053804 (5.42)	9	120749130	120 - 121	H3GA0054287 : H3GA0053804 (120.73 - 120.75)	120745879 - 120752715
	MARC0083358 (5.07)	9	122944920	122 - 123	MARC0083358 : MARC0073291 (122.94 - 123.46)	122937163 - 122944920
	ALGA0054777 (5.59)	9	127321307	127 - 128	ASGA0044392 : ALGA0054777 (127.05 - 127.32)	127291621 - 127321842
	ALGA0105115 (2.50)	9	148629187	148 - 149	ALGA0105115 : ASGA0088088 (148.63 - 148.64)	148613203 - 148631405
	H3GA0031644 (5.81)	11	25174659	25 - 26	H3GA0031644 : ASGA0050442 (25.17 - 25.25)	25172593 - 25179742
	ALGA0080254 (2.68)	14	107547829	107 - 108	MARC0051749 : DRGA0014395 (107.21 - 107.67)	107241709 - 107566295
	ALGA0085398 (4.17)	15	57805024	57 - 58	ASGA0069618 : ALGA0085400 (57.73 - 57.88)	57795466 - 57805831
**DFI**	ASGA0004976 (8.99)	1	176492950	176 - 177	DIAS0001368 : MARC0075909 (176.39 - 176.70)	176491002 - 176500481
	ALGA0006621 (10.15)	1	177818653	177 - 178	ASGA0004994 : INRA0004955 (177.76 - 178.25)	177818120 - 177825460
	INRA0004955 (10.15)	1	178254757	178 - 179	ASGA0004994 : INRA0004955 (177.76 - 178.25)	178248856 - 178263263
	MARC0013872 (9.66)	1	179327620	179 - 180	H3GA0003130 : H3GA0003149 (179.26 - 179.78)	179325282 - 179330134
	ALGA0009308 (3.71)	1	283682083	283 - 284	-	283676278 - 283690382
	H3GA0007369 (3.45)	2	118010721	118 - 119	MARC0011800 : ASGA0011273 (117.59 - 118.10)	118010721 - 118162933
	ALGA0029934 (4.59)	5	2658163	2 - 3	ASGA0095029 : M1GA0007255 (2.59 - 2.68)	2652808 - 2665244
	MARC0025903 (4.16)	9	53697784	53 - 54	H3GA0027334 : ASGA0043056 (53.60 - 53.89)	53687252 - 53704127
	ALGA0054797 (4.41)	9	128255464	128 - 129	ALGA0054797 : ASGA0044406 (128.26 - 128.29)	128242144 - 128257969
	ALGA0116599 (3.48)	12	103340	0 - 1	H3GA0055340 : MARC0003439 (0.08 - 0.98)	95807 - 115569
**DOT**	INRA0004895 (12.58)	1	176231881	176 - 177	ALGA0006570 : INRA0004895 (176.03 - 176.23)	176221961 - 176232960
	ASGA0004992 (11.07)	1	177744561	177 - 178	ALGA0006612 : ASGA0004992 (177.63 - 177.74)	177737575 - 177745406
	ALGA0006623 (11.11)	1	178024855	178 - 179	ASGA0004994 : INRA0004955 (177.76 - 178.25)	178024855 - 178030720
	INRA0004984 (13.28)	1	179188746	179 - 180	INRA0004984 : ASGA0005021 (179.19 - 179.24)	179185542 - 179190563
	H3GA0013527 (5.49)	4	102162642	102 - 103	ALGA0026864 : ALGA0026905 (101.98 - 102.71)	102160705 - 102168945
	MARC0012014 (4.95)	7	127278151	127 - 128	H3GA0023558 : MARC0012014 (126.78 - 127.28)	127242065 - 127278403
	ALGA0049934 (5.10)	8	141678271	141 - 142	ALGA0049934 : MARC0084432 (141.68 - 141.69)	141671480 - 141682377
	ASGA0042072 (4.99)	9	23437153	23 - 24	ASGA0042072 : DRGA0009221 (23.44 - 23.46)	23431543 - 23443051
	MARC0091244 (1.76)	13	12500465	12 - 13	MARC0071517 : ALGA0103574 (12.50 - 12.53)	12500225 - 12634473
**DFV**	ASGA0007897 (2.57)	1	303622361	303 - 304	ASGA0007890 : ASGA0007944 (303.61 - 303.69)	303619715 - 303637863
	ALGA0103394 (6.39)	6	105613840	105 - 106	ASGA0029217 : ALGA0103394 (105.55 - 105.61)	105610970 - 105617945
	MARC0035078 (4.58)	7	2509192	2 - 3	H3GA0019446 : MARC0035078 (2.49 - 2.51)	2507043 - 2518426
	H3GA0040087 (3.52)	14	50090206	50 - 51	ALGA0077324 : ASGA0063293 (47.53 - 51.33)	50085416 - 50940485
	ALGA0112899 (5.71)	16	8752527	8-9	-	8744303 - 8752527
**DFR**	H3GA0013527 (5.95)	4	102162642	102 - 103	ALGA0026864 : ALGA0026905 (101.98 - 102.71)	102161246 - 102166606
	H3GA0023563 (4.06)	7	127537686	127 - 128	H3GA0023563 : INRA0028862 (127.54 - 127.58)	127529470 - 127904678
	ASGA0039774 (7.20)	8	128899782	128 - 129	ALGA0049404 : ASGA0039774 (128.84 - 128.90)	128897970 - 128905934
	ALGA0081429 (4.05)	14	130733740	130 - 131	MARC0018296 : ASGA0066386 (130.57 - 130.96)	130711560 - 130790445
	MARC0085963 (5.71)	17	26443872	26 - 27	INRA0053116 : ALGA0093844 (25.80 - 26.84)	26438667 - 26445950
	MARC0055314 (3.00)	18	50389754	50 - 51	ASGA0080089 : MARC0033103 (50.36 - 50.62)	50325397 - 50389812
	H3GA0055497 (6.07)	X	109562447	109 - 110	ASGA0081247 : ALGA0099921 (109.15 - 110.38)	109557591 - 109569050
	H3GA0051891 (5.01)	X	110186817	110 - 111	ASGA0081247 : ALGA0099921 (109.15 - 110.38)	110186817 - 110195332

[1] FCR-feed conversion ratio, DFI-daily feed intake, DOT-daily occupation time, DFV-daily feeder visit, DFR-daily feeding rate.

[2] Single nucleotide polymorphism (SNP) that showed the highest significant association according to single-marker analysis in the 1-Mb genomic window.

[3] *Sus scrofa* chromosome according to genome build 10.2.

[4] Linkage disequilibrium (LD) blocks were defined using the ‘solid spine of LD’ algorithm implemented in the Haploview 4.2 software.

[5] The 95% confidence intervals were estimated according to Li (2011) (see text).
